# Using human rights measures to advance tobacco control- Japan and the Committee on the Elimination of Discrimination Against Women

**DOI:** 10.18332/tid/119140

**Published:** 2020-03-19

**Authors:** Action on Smoking and Health, Comité National Contre le Tabagisme, European Cancer Patient Coalition, Fiji Cancer Society, Global Bridges, Healis Sekhsaria Institute of Public Health, Indian Cancer Society- Delhi, International Alliance of Women, Japan Health Care Dental Association, Japan Society for Tobacco Control, Japanese Society of Cancer Nursing, OxySuisse, Pratyasha Anti-drugs Club, PROGGA, Public Health Law Center, Southeast Asia Tobacco Control Alliance, Society for Oral Cancer and Health, Swarna Hansa Foundation, Tobacco-Free Advocacy Japan, Tobacco - Free Association of Zambia, Policy Research for Development Alternatives

**Affiliations:** 1 Action on Smoking and Health, Washington, United States

**Keywords:** tobacco control, Japan, women

## Abstract

Japan has made progress on tobacco control in recent years. However, every year more than 157800 Japanese citizens1 and residents die from tobacco related diseases. Tobacco is a leading cause of preventable death and is therefore a considerable obstacle to the right to health of Japanese citizens. Tobacco is a human rights and women’s and girls’ rights issue and should be considered as part of the government’s human rights obligations. Tobacco, and the actions of the tobacco industry, prevents the women and girls of Japan from enjoying the highest attainable standard of health.

## RELEVANT FACTS AND STATISTICS

1. In Japan, more than 5.4 million women and 39200 girls smoke cigarettes each day^1^, and 4.88% of deaths among women in Japan are caused by tobacco.
2. Women and girls are also exposed to secondhand smoke (SHS), both at home and in public places, including work places. A recent study showed that the characteristics of participants exposed to SHS at home were: younger, women, those with lower educational attainment, lower income, living with a larger number of people in household, never smokers, and those with poor knowledge of tobacco’s adverse health effects^2^.
3. Of the 15000 deaths due to secondhand smoke annually in Japan, women account for roughly 10400 (over two-thirds) of the deaths”^3^.
4. The economic cost of smoking in Japan amounts to at least 4127905 million yen (about 37 billion dollars). This includes direct costs related to healthcare expenditures and indirect costs related to lost productivity due to early mortality and morbidity^1^.
5. Tobacco use also negatively impacts sustainable development and the environment, both of which have obvious negative consequences on the right to health of women and girls.
6. Families of smokers spend resources on cigarettes that could be spent on other household expenses. “A smoker in Japan would have to spend 1.85% of their average income (measured by per capita GDP) to purchase 10 of the most popular cigarettes to smoke daily each year.”^1^. The added healthcare expenses and lost income from tobacco induced-disease makes the financial situation much more dire.
7. Cigarette butts are the most commonly discarded pieces of waste worldwide. It is estimated that 81779 tons of butts which are not biodegradable and packs wind up as toxic trash in Japan each year^1^.
8. Approximately 5% of Japanese women smoke during pregnancy. Smoking during pregnancy is a cause of preterm delivery and impaired fetal growth. According to epidemiological estimates, pregnant women who smoke have almost double the risk of low birth weight and three times the risk of premature birth than pregnant women who do not smoke.
9. Japan Tobacco has about 61% of the market share in Japan. Japan’s Minister of Finance, in his official capacity, retains de facto control over the company holding 33.3% of the company as the single-largest shareholder^4^.

## SPECIAL CONCERNS

10. Women in the workforce
  - Even while Japan’s government has laudably expressed intentions and begun developing policies to improve circumstances for women in the workplace towards gender equality, tobacco use may be negatively impacting women’s participation in the workplace. Japan’s female labor force participation rate is approximately 51%5. For example, women, who are the primary home caregivers, may be forced to stay home to care for family members who are victims of tobacco related diseases.
  - Smoking is an impediment to the advancement of women in the workplace in Japan as only 7% of Japanese women smoke versus around 30% of men between the ages of 30 and 50 years. Approximately 30% of managers are smokers, which reflects the inequality in advancement as well as the inequality in health impact for women from exposure to secondhand smoke in the workplace6. The existence of smoking areas gives more access to managers by male smokers and also gives additional breaktime to male smokers.
  - Smoking, and desire to be free from exposure to secondhand smoke, could be a significant barrier for women in or trying to enter nontraditional roles in industries, which are highly male dominated like factories, transportation industry, construction industry and agriculture. It is not unusual for companies in such industries to have smoking rates of 50% or higher^6^. Even Toyota reports a smoking of 20% despite a plan to be tobacco-free by 2020^5^.
11. Failure to Inform
  - In regard to the exposure of women to secondhand smoke, the government of Japan has not provided sufficient warning on the health risk of secondhand smoke. The government should launch an education campaign, as well as spread this information through tobacco product package and carton warnings.
  - Current warnings have text that is hard to read because the lettering is small and in poor contrast to the color of the cigarette packages, and also use complicated and/or medical, or technical jargon that communicate poorly to diverse populations. Cartons have no warnings because they are currently exempted from health warning under Japanese laws.
12. Targeting of women
  - The tobacco industry targets women, specifically young women, with brands aimed at women with thin cigarettes and brightly colored tobacco product packages and promotional items for women (lighter holders, etc.), “low tar”, and flavored cigarettes including menthol^7^.
  - Tobacco companies are promoted as gender equal employers. For example, Japan Tobacco recently received Nadeshiko brand recognition for “encouraging women’s success in the workplace’’^8^.
13. Targeted advertising to the LGBT community
  - The tobacco industry has a history of targeted advertising to the LGBT community. LGBT (particularly LGBT smokers) are more likely to be exposed to and interact with tobacco-related messages on new and social media than their non-LGBT counterparts. Higher levels of tobacco media exposure were significantly associated with higher likelihood of tobacco use^9^.
  - In what is a clear conflict of interest, Japan Tobacco was recently recognized as “one of the most LGBT-friendly companies in Japan”^10^.

## LEGAL OBLIGATIONS

14. In 2018, Japan revised the Health Promotion Law (HPL). Under the HPL, in 2019, indoor areas of government offices and other public facilities including universities and hospitals became smoke-free. Controversially, outdoor smoking areas were permitted to continue in the premises of such offices and facilities.
15. Based on worker safety concerns that the HPL was not strong enough, the Tokyo Metropolitan Assembly passed the Tokyo Passive Smoking Prevention Ordinance. The HPL has wide exemptions for smoke-free requirements in existing restaurants and bars (exempting around 55% of such businesses entirely). However, the Tokyo ordinance exempts only restaurants and bars without employees. Thus, the ordinance will cover an estimated 84% of all restaurants and bars and has the potential to be a model for other local governments^11^.
16. Japan is Party to the World Health Organization’s Framework Convention on Tobacco Control (FCTC). The FCTC has been ratified by 180 countries and the European Union, which are obligated to put in place a range of measures to reduce tobacco use. The preamble encourages States Parties “to give priority to the right to protect public health”, and to respect the right of everyone to the enjoyment of the highest attainable standard of physical and mental health, as expressed in Article 12 of the International Covenant on Economic, Social and Cultural Rights (ICESCR).
17. The preamble of the FCTC also includes a statement “Recalling that the Convention on the Elimination of All Forms of Discrimination against Women, adopted by the United Nations General Assembly on 18 December 1979, provides that States Parties to that Convention shall take appropriate measures to eliminate discrimination against women in the field of health care”^12^.
18. In the preamble of the FCTC, Parties to the FCTC state that they are “Alarmed by the increase in smoking and other forms of tobacco consumption by women and young girls worldwide and keeping in mind the need for full participation of women at all levels of policy-making and implementation and the need for gender-specific tobacco control strategies”^12^.
19. The Japanese government has an obligation to “use its endeavors for the promotion and extension of social welfare and security, and of public health” under Article 25 of the Constitution of Japan and under Article 98 of the Constitution of Japan, treaties concluded by Japan such as the FCTC “shall be faithfully observed”^13^.

## RECOMMENDATIONS

We respectfully encourage CEDAW to call on the Japanese government to implement the obligations of the World Health Organization Framework Convention on Tobacco Control, including specifically,

20. Enact a law prohibiting smoking in all indoor public spaces, including workplaces, in line with its obligations under Article 8 of the FCTC and Article 25 of the Japanese constitution.
  - Article 8 Guidelines state that “The duty to protect from tobacco smoke, embodied in the text of Article 8, is grounded in fundamental human rights and freedoms. Given the dangers of breathing secondhand tobacco smoke, the duty to protect from tobacco smoke is implicit in, inter alia, the right to life and the right to the highest attainable standard of health, as recognized in many international legal instruments (including the Constitution of the World Health Organization, the Convention on the Rights of the Child, the Convention on the Elimination of all Forms of Discrimination against Women and the Covenant on Economic, Social and Cultural Rights), as formally incorporated into the preamble of the WHO Framework Convention and as recognized in the constitutions of many nations”.
  - Article 25 of the Japanese Constitution provides that “All people shall have the right to maintain the minimum standards of wholesome and cultured living. In all spheres of life, the State shall use its endeavors for the promotion and extension of social welfare and security, and of public health”.
  - The upcoming Olympics add to the sense of urgency around this issue: “the International Olympic Committee (IOC) demands not only that venues for the games be smoke-free, but that the entire host city be smoke-free. Since the dawn of the millennium every city except Tokyo that has held or will hold the Olympics has outlawed smoking in indoor public spaces, including Athens, Beijing and Pyeongchang, all of which are in countries with higher smoking rates than Japan’s”^14^.
21. Include the right to a smoke-free workplace in Act on Securing, Etc. of Equal Opportunity and Treatment between Men and Women in Employment (Act No. 113 of 1972) and the Basic Act for Gender Equal Society (Act No. 78 of 1999).
22. Ban all tobacco advertising, promotion, and sponsorship, as called for in Article 13 of the FCTC. This should include sports event sponsorship, generic company promotions, as well as a ban on product placements in media, whether direct payments are made to media companies or indirectly to individuals.
23. Raise tobacco taxes. The WHO benchmark is that a minimum of 70% of the final price of cigarettes consist of an excise tax. Japan’s tax is approximately 56%. Increasing tobacco taxes is very effective; every 10 per cent increase in the price of cigarettes reduces consumption by about 4 per cent among adults and about 7 per cent among youth. This has the additional benefit of increasing tax revenue for Japan, while simultaneously reducing the medical costs associated with tobacco use.
24. Provide pictorial warnings, especially for the risks of smoking and secondhand smoke for pregnant women and children, on tobacco product packages and cartons. Improvements in public education relating to tobacco including in school settings, plus public promotions. Public promotions should be done in efficacious manner such as cessation messaging in places where people smoke rather than brand advertising there.
25. Ban all flavored tobacco products such as menthol cigarettes and heated tobacco products.
26. Enact legislation explicitly allowing compensation of damages for damages to health and well-being of workers exposed to secondhand smoke.
27. Firmly exclude the tobacco industry from policy development, as required by FCTC Article 5.3.
28. Plain packaging or standardized packaging and restriction on use of bright colors like pink and purple and “thin” packages.
29. Provide, through national health insurance, smoking cessation approaches that address women’s concerns of depression and weight gain.

ASH US has been working with partners around the world to use human rights measures to advance tobacco control. While the WHO FCTC does not have a robust reporting and enforcement mechanism, many human rights treaties do, and often, the subject matter of those treaties overlaps with the goals of tobacco control. For example, ASH US and partners recently submitted a report on tobacco and its negative impact on the rights of women and girls in Japan to the Committee on the Elimination of Discrimination Against Women (CEDAW). This committee can use that report to question the Japanese government and encourage them to take steps to better protect their citizens from tobacco. If the government fails to protect their citizens from the tobacco industry, they will fail to live up to their obligations under international human rights law.
